# Hair Blanched by Sudden Fear

**Published:** 1851-10

**Authors:** 


### HAIR BLANCHED BY SUDDEN FEAR.

Our readers have all read of instances of this sort. We copy the following interesting case from the *Boston Medical and Surgical Journal*, or which it was reported by Dr. E. R. Smilie:

"A young man 23 years old came from the mines to San Francisco, with the intention of soon leaving the latter place for home. On the evening of his arrival, he, with his companions, visited the gambling saloons. After watching for a long time the varied fortunes of a table, supposed to be undergoing the process of "tapping," from the continued success of those betting against the bank, the excitement overthrew his better judgment, and he threw upon the "seven spot" of a new deal, a bag which he said contained 1100 dollars—*his all*, the result of two years' privation and hard labor—exclaiming, with a voice trembling from intense excitement, "*my home or the mines.*" As the dealer slowly resumed the drawing of his cards—with his countenance livid, from fear of the inevitable fate that seems ever attendant upon the tapping process when once commenced—I turned my eyes towards the young man who had staked his whole gains upon a card; and never shall I forget the impression made by his look of intense anxiety, as he watched the cards as they fell from the dealer's hands. All the energies of his system seemed concentrated in the fixed gaze of his eyes, while the deadly pallor of his face bespoke the subdued action of his heart. All around seemed infected with the sympathetic powers of the spell—even the hitherto successful winners forgot their own stakes, in the hazardous chance placed upon the issue of the bet. The cards are slowly told, with the precision of high wrought excitement. The seven spot wins! The spell is broken—re-action takes place. The winner exclaims, with a deep-drawn sigh, "*I will never gamble again,*" and was carried from the room in a deep swoon, from which he did not fully recover until the next morning, and then to know that the equivalent surrendered for his gain, was the color of his hair, now changed to a perfect white."

